# Epidemiological Distribution Characteristics of Tuberculosis Among Older Adults in Chongqing (2020-2024): Spatial-Temporal Analysis

**DOI:** 10.2196/89671

**Published:** 2026-05-05

**Authors:** Bojie Gao, Yu Xin, Wenping Liao, Lin Shi, Chengguo Wu, Jun Fan, Qingya Wang, Shanrong Huang, Xinyuan Yi, Yong Li, Wen Zhang, Chuan Pu

**Affiliations:** 1 School of Public Health, Chongqing Medical University, No 1, Yixueyuan Road, Chongqing, 400016, China, 86 18982276448; 2 Chongqing Institute of Tuberculosis Control and Prevention, Chongqing, Chongqing, China

**Keywords:** older adults, tuberculosis, epidemiology, spatial autocorrelation analysis, spatiotemporal clusters

## Abstract

**Background:**

With global aging, the burden of tuberculosis (TB) among older adults escalates, yet spatial studies on this group are scarce. In Chongqing, where 18.87% of the population are aged 65 years and older and TB burden is high, controlling older adult TB remains a major challenge.

**Objective:**

This study analyzed the spatiotemporal patterns of TB among adults aged 65 years and older in Chongqing, China, to inform local prevention and control strategies.

**Methods:**

The study data were obtained from the Tuberculosis Information Management System of China. Global and local spatial autocorrelation analyses were conducted using ArcGIS (version 10.7) to identify high-risk spatial clusters and visualize their distribution. Spatiotemporal scan statistics were performed using SaTScan (version 10.3.2) to detect clusters of TB cases among the older adult population. Statistical significance was set at *P<*.05.

**Results:**

The average annual incidence of TB among older adults in Chongqing was 69.59 per 100,000 population, with peaks occurring in spring and summer. The global Moran *I* ranged from 0.618 to 0.756 (*P*<.001 in all cases), indicating significant clustering. Persistent high-risk areas were identified in the northeastern and southeastern parts of Chongqing. Spatiotemporal scan statistics detected 1 most likely cluster (relative risk=3.52, 95% CI 3.37‐3.68; log-likelihood ratio=1017.43; *P*<.001) and 3 secondary clusters.

**Conclusions:**

Significant seasonal patterns of TB among older adults were observed in Chongqing. High-risk areas were predominantly concentrated in the northeastern and southeastern parts of the municipality. More targeted public health interventions are imperative.

## Introduction

Tuberculosis (TB), a chronic infectious disease primarily affecting the lungs, is caused by *Mycobacterium tuberculosis*. It ranks among the top 10 causes of death worldwide and represents a significant public health and social challenge worldwide [1]. According to the World Health Organization’s *Global Tuberculosis Report 2024* [1], an estimated 10.8 million individuals developed TB worldwide in 2023, resulting in approximately 1.25 million deaths. Among the 30 high–TB burden countries, China bears the third highest burden, with 741,000 new TB cases reported in 2023, accounting for 6.8% of the global incidence. Among these cases, 25,000 fatalities were attributed to TB. Although China has witnessed a declining trend in TB incidence over the years, the situation remains challenging due to its large population [2].

With increasing life expectancy and declining fertility rates, the pace of global population aging has accelerated markedly in the new century [3]. Older adults are more susceptible to TB due to immunosenescence, a higher prevalence of comorbid chronic diseases, and an increased risk of latent infection reactivation [4-6]. Consequently, the older adult population has become a critical challenge for TB control efforts. In the Western Pacific Region of the World Health Organization, the highest number of TB cases is observed in the age group of 65 years and older, largely driven by the demographic aging in China [1]. Located in southwestern China, Chongqing is a major populous municipality where the reported TB incidence has consistently ranked among the highest nationally. A study in Chongqing indicated that the average annual reported TB incidence from 2018 to 2022 was 65.01 per 100,000, exceeding the national average of 52.00 per 100,000 during the same period, with the peak incidence observed among individuals aged 65 years and older [1,7]. Another study reported that the spatial distribution of TB cases in Chongqing is heterogeneous, predominantly clustered in the northeastern and southeastern areas [8]. According to statistical data [9], the population aged 65 years and older in Chongqing reached 6.02 million by 2024, accounting for 18.87% of the municipality’s total population. Furthermore, TB management in older adults is compounded by challenges including delayed diagnosis, poor treatment adherence, and higher rates of adverse outcomes, presenting substantial obstacles to public health control efforts [10-12].

In recent years, spatiotemporal analysis has been widely used to characterize the distribution patterns and transmission dynamics of TB across space and time [13,14]. Studies conducted in China [15,16] and other countries [17-19] have demonstrated that TB exhibits highly complex dynamics with significant spatial heterogeneity at provincial, national, and international levels. The Chongqing municipality, characterized by its complex topography, significant urban-rural disparities, and frequent population mobility, demonstrates distinct spatial heterogeneity in its TB epidemic. However, few studies have investigated the spatiotemporal characteristics of TB distribution among the older adult population at the county level within Chongqing. Therefore, using reported TB data from 2020 to 2024 across Chongqing’s 38 counties, this study aimed to examine the geographical distribution and spatiotemporal trends of TB among individuals aged 65 years and older. The findings are expected to provide a scientific basis for targeted TB control, resource allocation, and policy formulation.

## Methods

### Overview of the Study Area

**Figure 1.**
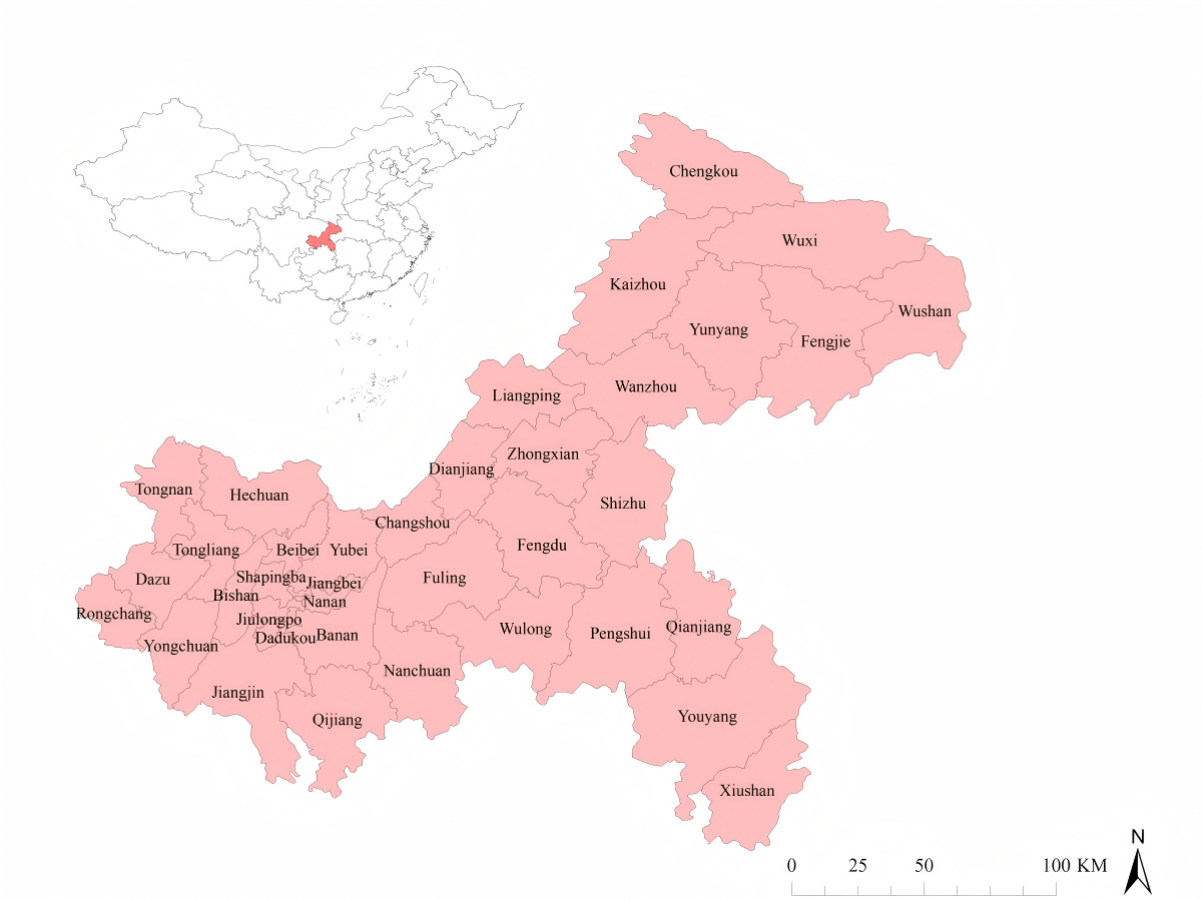
Geographical location and county-level administrative divisions of the Chongqing municipality, China.

Chongqing is located in the southwest of China in the upper reaches of the Yangtze River, between 28.10°N to 32.13°N and 105.11°E to 110.11°E, and is one of China’s 4 direct-controlled municipalities. It spans approximately 470 km from east to west and 450 km from north to south, covering a total area of 82,400 km^2^. The municipality comprises 38 counties, with a resident population of 31.90 million in 2024. Chongqing experiences a humid subtropical monsoon climate characterized by a mean annual temperature of 17.7 °C and an average relative humidity of 79.9% (Figure 1) [20].

### Data Collection and Management

The data for this study were obtained from the Tuberculosis Information Management System (TBIMS) of China, which collects case reports for all patients with TB during a specified period. TB incidence statistics were compiled based on patients’ current residential addresses. This study statistically analyzed the incidence of cases according to the patients’ current residences, collecting data on newly diagnosed older adult patients with TB aged 65 years and older reported in the system by medical institutions in the 38 counties of Chongqing from January 1, 2020, to December 31, 2024. To protect patient privacy and prevent duplicate reporting, each reported TB case was assigned a unique identifier. Each patient record contained demographic and clinical information, including age, sex, current residential address, patient type, diagnosis date, and diagnostic results. The diagnosis of TB followed the standards outlined in the WS 288-2017 guidelines issued by the National Health Commission of the People’s Republic of China [21] and the *Technical Guidelines for Tuberculosis Prevention and Control in China* [22]. From 2020 to 2024, population data for individuals aged 65 years and older in each county-level area of the Chongqing municipality were obtained from the records for the corresponding years in the 2024 Chongqing Statistical Yearbook [20] and the 2024 Chongqing Statistical Bulletin on National Economic and Social Development [9]. County-level administrative boundary vector maps for China and specifically for the Chongqing municipality were obtained from the National Platform for Common Geospatial Information Services. All spatial data were obtained in the WGS 84 geographic coordinate system.

### Spatial Trend Surface Analysis

Trend surface analysis uses a 3D coordinate system (*x*, *y*, *z*) to generate a 3D projection of the data, thereby graphically illustrating the spatial distribution trends of TB. In this coordinate system, the x- and y-axes correspond to the longitude and latitude of the geometric center of each study unit, respectively, whereas the z-axis represents the incidence rate. A higher *z* value indicates a greater TB incidence in the corresponding area. Thus, any point (*x*, *y*, or *z*) within this 3D space corresponds to the TB incidence level within a specific study region. By projecting the longitude and latitude coordinates of each county in Chongqing and their corresponding TB incidence rates onto the *x*-*z* and *y*-*z* planes and subsequently performing curve fitting using a polynomial model [23], the characteristics of incidence rate trends in relation to geographical coordinates can be further analyzed in 2D.

### Spatial Autocorrelation Analysis

Spatial autocorrelation measures the correlation of a single variable across different spatial locations, quantifying the degree of clustering for an attribute value among spatial units. It is typically categorized into global and local spatial autocorrelation analyses. This study used the global Moran *I* index to assess global spatial autocorrelation. The value of the Moran *I* ranges from −1 to 1. A Moran *I* value greater than 0 indicates positive spatial autocorrelation, with higher values signifying more pronounced spatial clustering. Conversely, a Moran *I* value of less than 0 indicates negative spatial autocorrelation, suggesting a dispersed pattern; values closer to −1 denote greater spatial disparity. A Moran *I* of 0 implies the absence of spatial clustering, consistent with a random distribution [24]. The statistical significance was evaluated using the standardized *z* score.

Global spatial autocorrelation was used to determine whether TB incidence was correlated across the entire study area, but it cannot pinpoint the specific locations of clusters. In contrast, local spatial autocorrelation analysis, conducted by generating cluster maps based on local indicators of spatial association (LISA), measures the concentration of high or low values. This method detects spatial hot spots and cold spots of disease occurrence, thereby revealing spatial heterogeneity within the data. The LISA cluster patterns are categorized into 4 types: high-high, high-low, low-high, and low-low. Among these, high-high and low-low clusters represent positive spatial autocorrelation, whereas high-low and low-high clusters indicate negative spatial autocorrelation. The high-high clusters, identified as hot spots, are typically prioritized areas for infectious disease control and were the primary focus of the spatial analysis of TB in the older adult population [25]. To assess robustness, we excluded 8 counties with populations of less than 100,000 (Dadukou, Yuzhong, Jiangbei, Nan’an, Liangping, Jiulongpo, Tongnan, and Shapingba) and reran LISA for 2020 to 2024 using the remaining 30 counties.

In this study, the spatial weight matrix for spatial autocorrelation analysis was constructed using the inverse distance method, with weights calculated as the reciprocal of the Euclidean distance between the centroids of each district and county. No distance threshold was set, meaning that all districts and counties were considered neighbors, and the weight matrix was row standardized before analysis.

### Spatial-Temporal Scan Statistic

Spatiotemporal scan statistics were used to identify spatial clusters of TB incidence among older adults in Chongqing and analyze their temporal evolution. The analysis was performed using the spatiotemporal scan statistic by Kulldorff [26], with a Poisson probability model applied to the number of reported TB cases, resident population data, and geographical coordinates. In this method, the scanning window is a dynamic cylinder whose base represents a circular geographic area and whose height corresponds to a time interval. As the center, radius of the base, and height of the cylinder vary, a log-likelihood ratio (LLR) is computed for each possible window to compare the risk of TB inside the window against the risk outside. The relative risk (RR) was calculated to estimate the magnitude of risk within each identified cluster. The 95% CIs for the RR were derived using the exact Poisson method based on the observed and expected case counts. The statistical significance of the LLR statistic was assessed using the Monte Carlo randomization method, with 999 iterations performed to derive the *P* value. A larger, statistically significant LLR value indicates a stronger clustering effect. The window with the maximum significant LLR was identified as the most likely cluster, whereas other windows with statistically significant LLRs were classified as secondary clusters. For this study, the scan period spanned January 1, 2020, to December 31, 2024, with 1 year defined as the basic temporal unit. The maximum spatial and temporal scan window sizes were both set to the default values, with the maximum spatial cluster size set to 50% of the at-risk population and the maximum temporal cluster size set to 50% of the study period to ensure that persistent aggregation phenomena could be detected and that the reported time range reflected the actual duration of increased risk [27]. Clusters were ranked based on their LLR values. The geographic coordinates (longitude and latitude) used for spatiotemporal scanning were the centroid coordinates of each county-level administrative district in Chongqing obtained from the National Geographic Information Public Service Platform of China. These coordinates represent the spatial location of each district for aggregated case data analysis rather than individual patient addresses.

### Statistical Software

Data collection, collation, and descriptive analysis for TB cases among individuals aged 65 years and older were performed using Microsoft Excel 2019. Joinpoint regression analysis (version 6.0.0.0; National Cancer Institute) was used to assess temporal trends in TB notification rates. Global and local spatial autocorrelation analyses, including the calculation of the Moran *I* index, spatial trend surface analysis, and visualization of incidence rates, were conducted using the ArcGIS software (version 10.7; Esri). Spatiotemporal scanning and the calculation of LLR values were performed using the SaTScan software (version 10.3.2). A threshold of *P<*.05 was applied to determine statistical significance for all analyses.

### Ethical Considerations

The use of TB case data in this study was approved by the ethics committee of the Chongqing Tuberculosis Control and Prevention Institute (audit 2025008) on April 14, 2025. This was a retrospective approval for the use of deidentified data collected from January 1, 2020, to December 31, 2024, from the routine TBIMS. The ethics committee waived the requirement for individual informed consent because the data were anonymized prior to analysis. The authors have obtained formal permission to access and use the data for this research. All personal identifiers were removed from the dataset prior to data analysis and reporting.

## Results

### Descriptive Analysis of TB Cases

**Figure 2.**
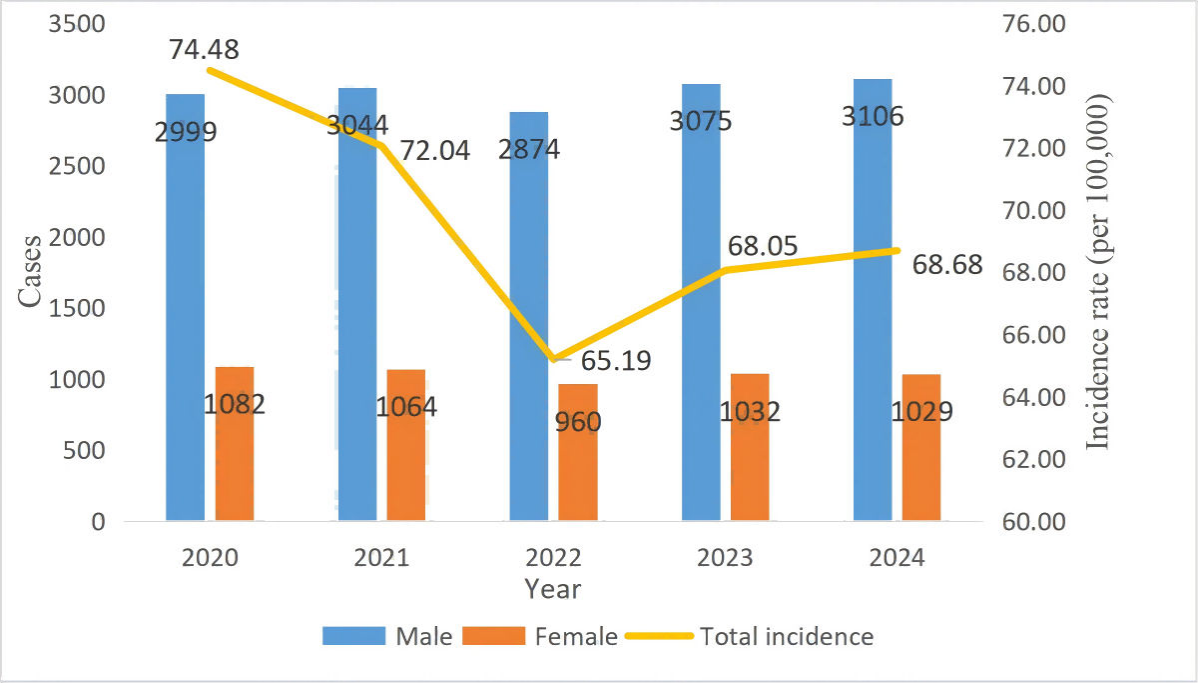
Number of cases and overall notification rate of tuberculosis among older adults by sex in the Chongqing municipality, 2020 to 2024.

**Figure 3.**
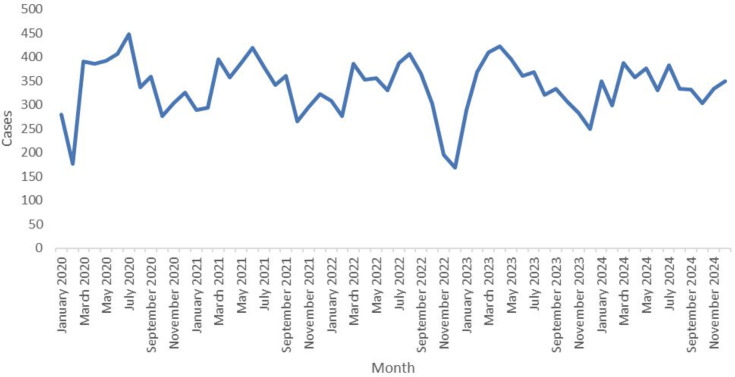
Monthly distribution of tuberculosis cases among older adults in the Chongqing municipality, 2020 to 2024.

A total of 20,265 new TB cases in individuals aged 65 years and older were reported from 2020 to 2024, yielding an average of 4053 cases annually. Among these 20,265 patients, 15,098 (74.5%) were male, and 5167 (25.5%) were female, resulting in a male-to-female ratio of 2.92:1. The average annual incidence rate was 69.59 per 100,000 population. The specific annual rates were 74.48, 72.04, 65.19, 68.05, and 68.68 per 100,000 population in 2020, 2021, 2022, 2023, and 2024 (Figure 2). Joinpoint regression analysis identified a statistically significant change in trend occurring in 2022. From 2020 to 2022, the notification rate decreased sharply, with an annual percent change (APC) of −6.26% (95% CI −9.94% to −2.09%; *P*<.001). From 2023 to 2024, the rate increased slightly, with an APC of 2.11% (95% CI −2.31% to 6.43%; *P*=.28). The average APC over the entire 2020 to 2024 period was −2.17% (95% CI –5.05% to 0.72%; *P*=.16), indicating that the overall incidence did not change significantly over the 5 years. Regarding age distribution, 35.99% (7293/20,265) of the patients were aged 65 to 69 years, 30.22% (6125/20,265) were aged 70 to 74 years, and 33.79% (6847/20,265) were aged 75 years or older. Patients were mainly referred, accounting for 71.22% (14,432/20,265) of the total, followed by direct visits with 12.22% (2476/20,265) and tracking with 11.56% (2343/20,265; Table 1). From 2020 to 2024, the number of TB cases showed seasonal variation, with the peak of reported cases mainly concentrated in spring and summer and a small peak occurring every year in March (Figure 3).

**Table 1.** Epidemiological characteristics of tuberculosis among older adults in Chongqing, 2020 to 2024 (N=20,265).

Characteristic	Patients, n (%)
Sex	
Male	15,098 (74.5)
Female	5167 (25.5)
Age group (y)	
65-69	7293 (35.99)
70-74	6125 (30.22)
≥75	6847 (33.79)
Patient sources	
Health examination	51 (0.25)
Recommended due to symptoms	526 (2.6)
Direct consultation	2476 (12.22)
Active screening	436 (2.15)
Referral	14,432 (71.22)
Tracking	2343 (11.56)
Other	1 (0)

### Spatial Characteristics of TB Cases

**Figure 4.**
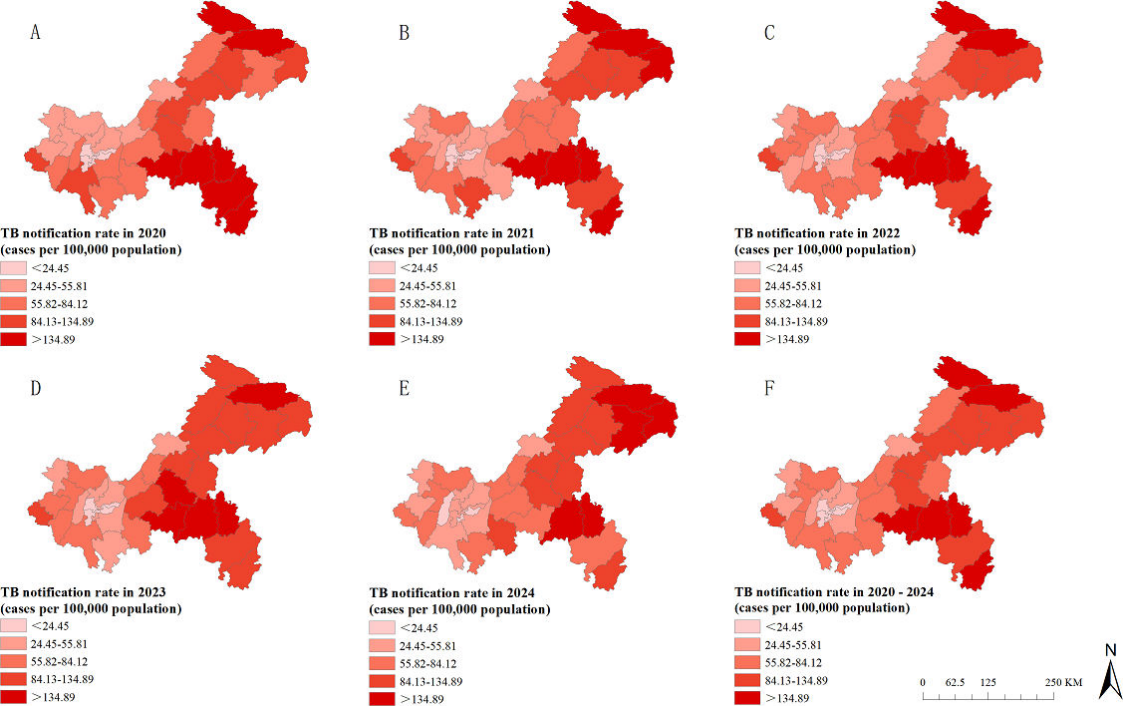
Spatial distribution map of tuberculosis (TB) notification rates among the older adult population in the Chongqing municipality, 2020 to 2024.

Spatial distribution maps of reported TB incidence rates for the 38 counties in Chongqing (2020‐2024) were generated using ArcGIS. From a spatial perspective, the incidence of TB was mainly distributed in the northeast and southeast of Chongqing. The top 5 regions with the highest reported TB incidence rates among individuals aged 65 years and older were Pengshui (255.38 per 100,000 population), Qianjiang (192.33 per 100,000 population), Wuxi (180.20 per 100,000 population), Wulong (175.44 per 100,000 population), and Xiushan (174.26 per 100,000 population; Figure 4).

### Spatial Trend Surface Analysis

**Figure 5.**
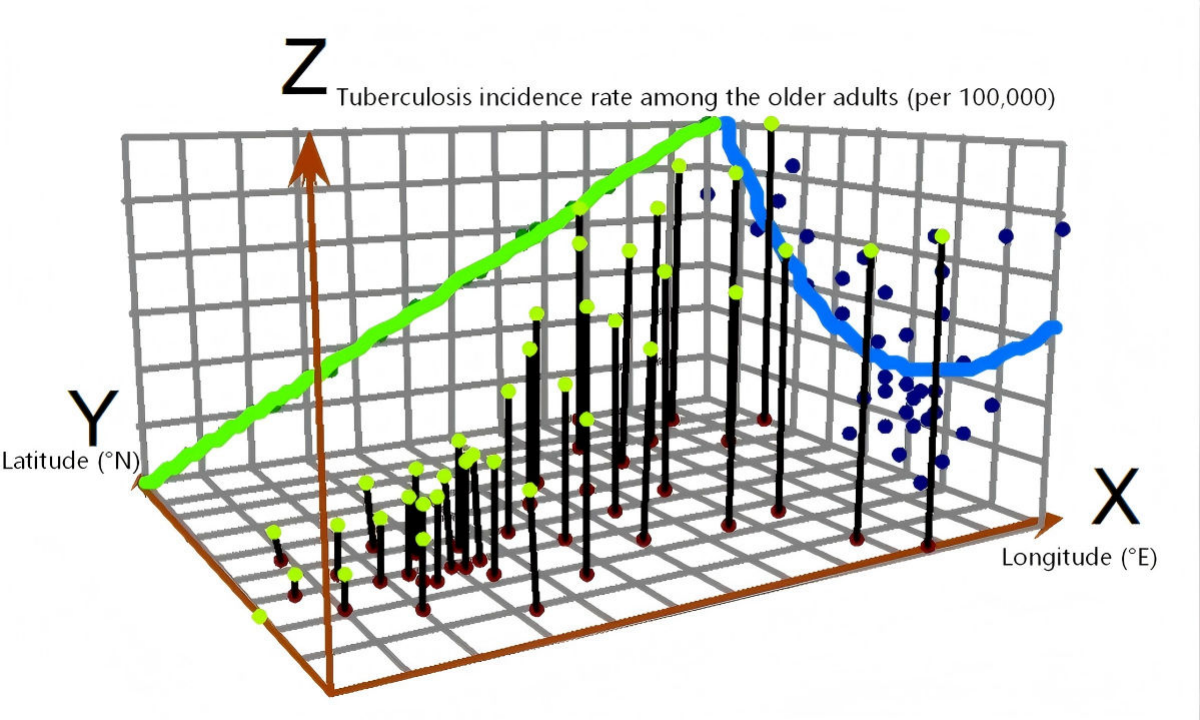
Spatial 3D trend surface analysis of tuberculosis among older adults in the Chongqing municipality, 2020 to 2024.

Using the reported incidence rate of TB among older adults in the 38 counties of Chongqing from 2020 to 2024 as the dependent variable (z-axis) and the latitude and longitude of the geometric center of the study area as the independent variables (x- and y-axes), the analysis revealed a clear east-west gradient, with incidence rates gradually increasing from west to east. In the north-south direction, the rates exhibited a U-shaped pattern, initially decreasing from north to south before rising rapidly further south. Overall, the trend surface indicated higher incidence rates in the eastern and northern regions than in the western and southern parts of the municipality (Figure 5).

### Spatial Autocorrelation Analysis

The results of the global spatial autocorrelation analysis of reported TB incidence among older adults in Chongqing from 2020 to 2024 showed that the global Moran *I* indexes were 0.636, 0.618, 0.756, 0.713, and 0.668, respectively, with all Moran *I* values greater than 0 and statistically significant (*P*<.001; Table 2). This indicates a positive global spatial autocorrelation in TB incidence from 2020 to 2024, meaning that there was spatial clustering. The spatial clustering of TB in older adults was highest in 2022.

**Table 2.** Global spatial autocorrelation analysis of tuberculosis notification rates among older adults in the Chongqing municipality, 2020 to 2024.

Year	Moran *I*	*z* score	*P* value
2020	0.636	6.613	<.001
2021	0.618	6.560	<.001
2022	0.756	7.476	<.001
2023	0.713	7.086	<.001
2024	0.668	6.844	<.001

**Figure 6.**
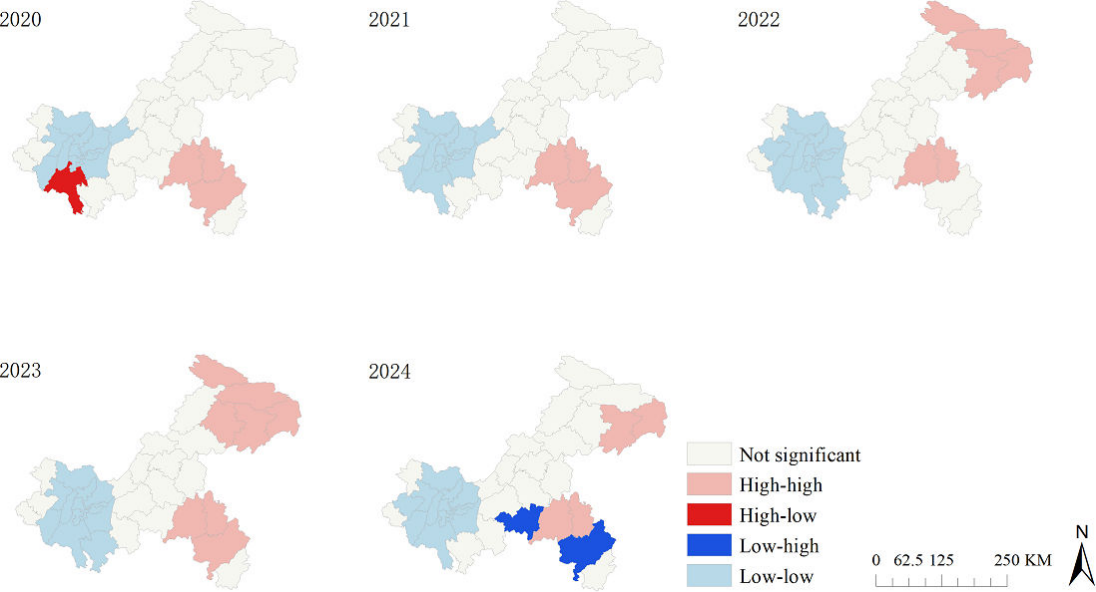
Local spatial autocorrelation analysis of tuberculosis notification rates among older adults in the Chongqing municipality, 2020 to 2024.

The LISA cluster maps from the local spatial autocorrelation analysis for the period from 2020 to 2024 revealed 4 types of spatial clustering patterns: high-high, high-low, low-high, and low-low clusters. Specifically, no low-high clusters were identified in 2020, neither high-low nor low-high clusters were observed from 2021 to 2023, and no high-low clusters were found in 2024. In 2020 and 2021, high-high clusters were located in the southeastern region, including Pengshui, Qianjiang, and Youyang. Starting in 2022, the high-high clusters expanded from the southeast to the northeast, encompassing Pengshui, Qianjiang, Youyang, Wushan, Fengjie, Wuxi, Chengkou, and Yunyang. Throughout the entire study period, Pengshui and Qianjiang in southeastern Chongqing consistently remained hot spots for TB among older adults. Cold spots were primarily distributed in the central urban districts and their surrounding counties (Figure 6).

The sensitivity analysis results show that the southeastern hot spots (Pengshui and Qianjiang) remained stable; northeastern hot spots began to appear in 2022, which is almost consistent with the LISA cluster patterns shown in Figure 6, in which high-high clusters were concentrated in southeastern Chongqing and expanded to the northeast after 2022. The counties that were excluded were all cold spots and had no impact on the core results (Multimedia Appendix 1).

### Spatial-Temporal Clustering Analysis Using SaTScan

The spatiotemporal scan analysis identified 1 most likely cluster and 3 secondary clusters of reported TB incidence among older adults in Chongqing from 2020 to 2024. The most likely cluster covered 5 counties (Xiushan, Youyang, Qianjiang, Pengshui, and Wulong) in the southeastern region, with a clustering period from January 2020 to December 2021. This cluster contained 1970 confirmed cases (RR=3.52, 95% CI 3.37-3.68; LLR=1017.43; *P*<.001), indicating the highest risk of TB among older adults in these areas. Secondary cluster 1 encompassed 17 counties (Wanzhou, Yunyang, Zhongxian, Kaizhou, Liangping, Shizhu, Fengjie, Dianjiang, Fengdu, Wuxi, Chengkou, Qianjiang, Changshou, Wushan, Pengshui, Fuling, and Wulong) primarily distributed across the northeastern and southeastern regions. Its clustering period was from January 2023 to December 2024, involving 4623 cases (RR=1.70, 95% CI 1.65-1.75; LLR=454.45; *P*<.001). Secondary cluster 2 included 3 counties (Yongchuan, Jiangjin, and Rongchang) and was observed from January 2020 to December 2021, with 924 cases identified (RR=1.21, 95% CI 1.13-1.29; LLR=14.55; *P*<.001). Secondary cluster 3 covered a single county, Qijiang, during the period from January 2020 to December 2021, with 312 cases (RR=1.27, 95% CI 1.14-1.42; LLR=8.31; *P*=.03; Table 3). It is worth noting that the detected clusters span multiyear periods (2020‐2021 and 2023‐2024) rather than single years. This is because the spatiotemporal scan analysis allowed for a maximum temporal cluster size of 50% of the study period (ie, 2.5 years), a standard setting that enables the identification of both short-term and persistent aggregations. Therefore, the 2-year duration of these clusters reflects sustained elevated risk in the affected regions rather than isolated annual events.

**Table 3.** Spatiotemporal scan cluster analysis of tuberculosis cases among older adults in the Chongqing municipality, 2020 to 2024.

	Most likely cluster	Secondary cluster 1	Secondary cluster 2	Secondary cluster 3
Time frame	January 1, 2020, to December 31, 2021	January 1, 2023, to December 31, 2024	January 1, 2020, to December 31, 2021	January 1, 2020, to December 31, 2021
Cluster districts and counties	Xiushan, Youyang, Qianjiang, Pengshui, and Wulong	Wanzhou, Yunyang, Zhongxian, Kaizhou, Liangping, Shizhu, Fengjie, Dianjiang, Fengdu, Wuxi, Chengkou, Qianjiang, Changshou, Wushan, Pengshui, Fuling, and Wulong	Yongchuan, Jiangjing, and Rongchang	Qijiang
Center/radius	28.49°N, 109.01°E/160.58 km	30.71°N, 108.40°E/162.39 km	29.29°N, 105.87°E/40.42 km	28.88°N, 106.72°E/0 km
Observed cases, n	1970	4623	924	312
Expected cases	601.49	3000.75	772.53	245.81
LLR^a^	1017.43	454.45	14.55	8.31
RR^b^ (95% CI)	3.52 (3.37-3.68)	1.70 (1.65‐1.75)	1.21 (1.13‐1.29)	1.27 (1.14‐1.42)
*P* value	<.001	<.001	<.001	.03

a LLR: log-likelihood ratio.

b RR: relative risk.

## Discussion

### Principal Findings

This study used spatial epidemiological methods to analyze the spatiotemporal characteristics of reported TB incidence among the population aged 65 years and older across 38 counties of Chongqing from 2020 to 2024, thereby delineating the epidemiological distribution of TB within this demographic.

This study found that, from 2020 to 2024, the average annual incidence rate among older people in Chongqing was 69.59 per 100,000 population, higher than the average TB incidence rate in China of 52.00 per 100,000 population [1] and the annual incidence rate of TB among people aged 65 years and older in Xuzhou of 51.27 per 100,000 population [28]. Furthermore, joinpoint regression analysis revealed a nonlinear trend in TB incidence among older adults in Chongqing, with a significant decline from 2020 to 2022 followed by a nonsignificant increase from 2023 to 2024. The average APC over the entire study period was −2.17% (95% CI −5.05% to 0.72%; *P*=.16). Although this decline did not reach statistical significance, the point estimate was substantially lower than the reported national average annual decline of −3.55% for older adult TB in China [29]. This finding may be attributable to Chongqing’s high aging population, with 18.87% of people aged 65 years and older, which is higher than the national average of 15.40% [30]. Moreover, Chongqing is mostly mountainous, and the aging and empty nest phenomena are serious, resulting in lower health awareness among the local older adults left behind and a lack of knowledge about TB prevention and control [31]. Despite the nonlinear pattern identified by the joinpoint analysis, the overall incidence among older adults in Chongqing decreased from 74.48 per 100,000 population in 2020 to 68.68 per 100,000 population in 2024, a net decline consistent with observations from other Chinese provinces [32,33] and other countries [34,35]. The overall declining trend may be attributed to factors such as national socioeconomic development, improvements in health care infrastructure, and the implementation of effective public health interventions [36,37]. Concurrently, this trend aligns with a series of TB control policies enacted by the Chongqing municipal government and health administration. For instance, the Chongqing Action Plan for Tuberculosis Control (2019-2022) issued in 2019 [38] promoted initiatives including the Hundreds, Thousands, and Tens of Thousands of Volunteers campaign for TB knowledge dissemination, the integration of TB education into school health programs, and the comprehensive incorporation of TB control for migrant populations into localized management. These measures ensured seamless processes from screening and treatment to cross-regional coordination.

The turning point detected in 2022 by the joinpoint analysis closely mirrors the trajectory of the COVID-19 pandemic. It is important to acknowledge that the COVID-19 pandemic introduced significant disruptions to TB surveillance worldwide, which may have influenced case detection and reporting during our study period. A growing body of literature has documented the substantial impact of the pandemic on TB notifications worldwide. According to the World Health Organization, TB case notifications fell considerably below prepandemic levels from 2020 to 2021, with a global reduction of 18% in 2020 [39]. Studies from Brazil reported significant decreases in TB notifications in 2020 (13%) and 2021 (9%), with partial recovery in 2022 [40]. Similar disruptions were observed across multiple countries, attributed to health care system strain, reallocation of TB diagnostic resources and personnel to COVID-19 response, lockdown measures that limited health care access, and reduced health care–seeking behavior due to fear of infection [41,42]. The overlap of respiratory symptoms between TB and COVID-19 may also have contributed to diagnostic delays and underreporting [43].

In the context of these global disruptions, it is noteworthy that large-scale preventive measures implemented in China during the pandemic, such as disinfection and isolation protocols, may have helped limit TB transmission [44]. Simultaneously, reduced social activities and increased adherence to protective measures such as mask wearing among older adults likely diminished TB exposure risks [45]. These factors may have partially offset the surveillance disruptions observed elsewhere. However, the brief upward trend from 2022 to 2024 requires further consideration. This increase may be related to the relaxation of pandemic restrictions in China after 2022, which led to increased outdoor activities among older adults [46]. Additionally, some older adults who had been infected with COVID-19 may have experienced temporary immune impairment [47], potentially increasing their susceptibility to TB. This combination of behavioral and immunological factors likely contributed to the observed temporary increase against the backdrop of ongoing postpandemic recovery in health care use.

A marked male predominance was observed in TB cases among older adults, a finding consistent with those of numerous previous studies [48,49]. This disparity is primarily attributed to gender-specific differences in lifestyle behaviors and genetic susceptibility [50,51]. High-risk behaviors more prevalent in male individuals, such as smoking, alcohol consumption, and poorer adherence to medical care, can increase susceptibility to TB infection. Concurrently, male individuals may exhibit a higher probability of specific genetic variations that elevate the risk of contracting TB. The predominant patient source being referral was likely because older adults are a key population for TB control. When primary health care institutions detect suspected patients, they are typically transferred to designated TB facilities to mitigate the risk of clustered transmission [52]. Furthermore, our analysis revealed a distinct seasonal variation in TB incidence among older adults in Chongqing, with peaks predominantly occurring in spring and summer. This pattern aligns with observations from multiple regions, including the Hong Kong special administrative region, China [53], Guangxi [54], Turkey [55], and Japan [56]. This seasonality may be associated with care seeking delays among Chinese older adults. This may be related to delayed medical visits among older people in China. Winter in China is from December to February, during which people prepare to celebrate the Chinese New Year. Some patients with mild symptoms may choose to seek medical treatment after the New Year; therefore, medical visits are avoided or postponed during this period, leading to a decrease in the reported number of cases [57].

The spatial analysis confirmed that TB among older adults in Chongqing is not randomly distributed but exhibits significant clustering. The identification of persistent hot spots in both southeastern and northeastern regions aligns with areas previously characterized by mountainous terrain, ethnic minority populations, and limited health care access [58]. These geographic and socioeconomic factors likely contribute to delayed diagnosis and ongoing transmission, particularly among older adult residents with lower health literacy [8,20].

Sensitivity analysis revealed marked regional heterogeneity in the robustness of these spatial patterns. Southeastern hot spots (eg, Pengshui and Qianjiang) demonstrated exceptional stability, persisting even after excluding low-population counties, suggesting that risk in this region is driven by entrenched, population-independent factors. In contrast, northeastern hot spots exhibited greater temporal variability; although the expansion of risk to the northeast since 2022 was reproduced in the sensitivity analysis, the detection of individual counties was more sensitive to spatial weight structures. These findings have practical implications for TB control. The southeastern region is highly stable and requires continuous, population-wide interventions, including enhanced active case detection, routine screening, and health education campaigns [59,60]. However, the northeastern region experiences considerable fluctuations in the epidemic and requires localized strategies, such as conducting large-scale screenings regularly in newly identified high-incidence counties and detecting new cluster cases through real-time monitoring. To further investigate the spatiotemporal clustering of reported TB incidence among older adults in Chongqing and delineate the specific scope and size of the clusters, this study conducted a spatiotemporal scan analysis. The results identified the most likely cluster in 5 southeastern counties: Xiushan, Youyang, Qianjiang, Pengshui, and Wulong. Secondary clusters were distributed across 17 districts and counties in both the northeastern and southeastern regions. These findings are consistent with the results of the spatial analyses. No significant clusters were detected in 2022, which may reflect pandemic-related fluctuations in case detection or a more dispersed spatial distribution during that year. Therefore, for future prevention and control efforts, it is imperative to sustain and enhance surveillance and interventions in these key areas. TB control strategies should be adjusted and refined according to the specific clustering patterns observed in Chongqing to effectively reduce the risk of TB transmission and the overall disease burden.

### Limitations

Nevertheless, this study has several limitations. First, the data were derived from passively reported TB cases, which may not capture all incidents due to underreporting. In some remote areas, factors such as limited mobility among older adults and insufficient diagnostic capacity in primary health care institutions could lead to missed cases and incomplete registration. Second, the research period (2020‐2024) largely overlapped with the COVID-19 pandemic, which may have affected TB case reporting through multiple pathways, such as health care system pressures, lockdown measures, and reduced health care–seeking behavior [3,4]. Although our data came from the mandatory national TB reporting system (TBIMS) with standardized procedures, we cannot rule out a certain degree of underreporting or diagnostic delays during the peak of the pandemic (2020-2022). At the same time, several pandemic-related factors may have introduced difficult-to-quantify confounding effects. The potential impact of these confounding effects on the spatiotemporal distribution characteristics also cannot be entirely ruled out. Despite these potential impacts, the spatiotemporal distribution patterns we identified—particularly the persistent clustering in the northeast and southeast regions of Chongqing—are consistent with prepandemic research results, indicating that the underlying epidemiological patterns remain valid. Future studies that integrate multisource data, such as active surveillance data, postpandemic follow-up surveys, or health care use data, will help verify trends in true incidence and further clarify the confounding effects caused by the pandemic. Third, the absence of detailed geographical information at the township level for each county precluded a finer-scale spatiotemporal cluster analysis. Finally, as the older adult population is highly vulnerable to TB, its incidence is influenced by multiple factors, including socioeconomic status, health care accessibility, climatic conditions, and the intensity of policy support. The specific impacts of these factors on TB in older adults warrant further investigation.

### Conclusions

This study delineated the spatiotemporal distribution patterns and clustering characteristics of TB among individuals aged 65 years and older in Chongqing from 2020 to 2024. The incidence of TB among the older adult population showed a significant downward trend from 2020 to 2022 and no significant change from 2023 to 2024, with cases predominantly observed in older men and peaks occurring during spring and summer. Spatial analysis identified persistent high-cluster areas in both the northeastern and southeastern regions of Chongqing. Spatiotemporal scan analysis further indicated a higher TB burden and increased transmission risk in the southeastern part of the municipality. Therefore, it is crucial to sustain and strengthen TB control measures in these high-risk areas by adopting more targeted outbreak containment strategies, enhancing the diagnostic and treatment capabilities of health care professionals, and reducing underreporting of TB cases among older adults.

## Supplementary material

10.2196/89671 Multimedia Appendix 1 Sensitivity analysis of tuberculosis in the older adult population in Chongqing from 2020 to 2024 based on population thresholds.

## Abbreviations

APC: annual percent change
LISA: local indicators of spatial association
LLR: log-likelihood ratio
RR: relative risk
TB: tuberculosis
TBIMS: Tuberculosis Information Management System
