# Supplementary figures and images for: Epidemiological Distribution Characteristics of Tuberculosis Among Older Adults in Chongqing (2020-2024): Spatial-Temporal Analysis
Source: JMIR Public Health Surveill. 2026 May 5;12:e89671. doi: 10.2196/89671 (PMC13143199; doi:10.2196/89671)

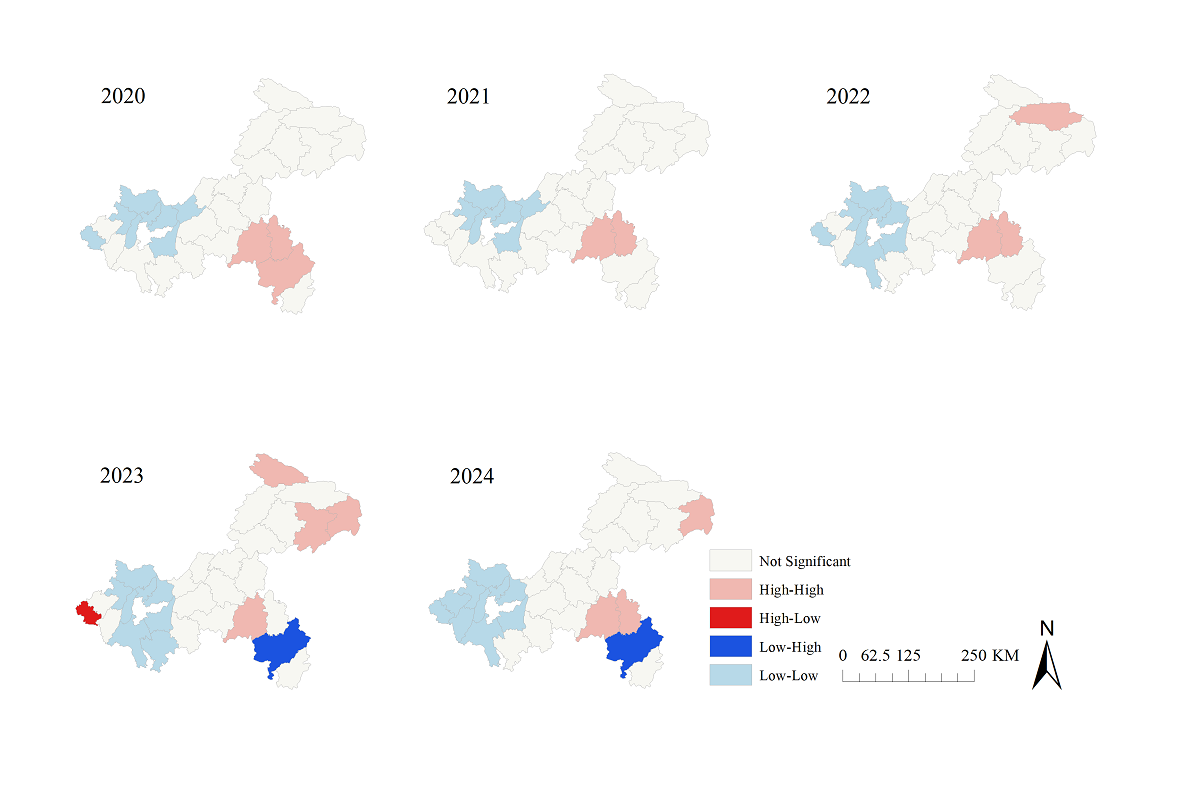

Supplement: Multimedia Appendix 1 [file publichealth-v12-e89671-s001.png]
